# Supplementary material for: An Analysis of the Possible Migration Routes of Oedaleus decorus asiaticus Bey-Bienko (Orthoptera: Acrididae) from Mongolia to China
Source: Insects. 2022 Jan 10;13(1):72. doi: 10.3390/insects13010072 (PMC8781545; doi:10.3390/insects13010072)
Supplement: Supplementary file 1 [file insects-13-00072-s001.zip › insects-1474041-supplementary.pdf]

Table S1. The meteorological systems in the immigration peaks in Yanqing from 2012 to 2017.

Peak Period	Landing Factor			
	**Wind Shear**	**Downdraft**	**Low Temperature Barrier**	**Precipitation**
19 July 2012	+	+		
20 July 2012				
23 July 2012				
26 July 2012				+
04 August 2012				
22 August 2012		+		
08 July 2013	+			+
16 July 2013				+
17 July 2013				
18 July 2013	+			+
28 July 2013		+		
10 August 2013				
12 August 2013				+
14 August 2013				
15 August 2013	+			
16 August 2013	+	+		
24 August 2013		+		
30 June 2014				+
19 July 2014				
20 July 2014	+			
21 July 2014		+		+
02 August 2014				
03 August 2014	+	+		
11 August 2014		+		
19 August 2014	+			
13 July 2015	+	+		
15 July 2015	+	+		+
16 July 2015				+
22 July 2015	+			+
26 July 2015		+		
28 July 2015		+		+
14 August 2015		+		
15 August 2015	+	+		
21 August 2015	+	+		+
24 August 2015				+
04 September 2015		+		+
26 July 2016				
29 July 2016				+

08 July 2017	+	+	
09 July 2017	+	+	
11 July 2017		+	
12 July 2017		+	
16 July 2017			
18 July 2017			+
19 July 2017			
20 July 2017	+	+	+
29 July 2017			
03 August 2017		+	
07 August 2017	+	+	
11 August 2017		+	+
12 August 2017			+
13 August 2017			+
14 August 2017		+	
15 August 2017	+	+	+
16 August 2017	+	+	+
17 August 2017		+	
18 August 2017			+
19 August 2017		+	+
20 August 2017		+	
21 August 2017	+	+	
22 August 2017	+		+
23 August 2017			
31 August 2017	+		
01 September 2017			
02 September 2017			
04 September 2017			
08 September 2017			
09 September 2017	+		
11 September 2017	+		

Note: + The factor bringing out immigration peaks of *Oedaleus asiaticus*.
